# Leucine Supplementation in Cancer Cachexia: Mechanisms and a Review of the Pre-Clinical Literature

**DOI:** 10.3390/nu14142824

**Published:** 2022-07-09

**Authors:** Anna G. Beaudry, Michelle L. Law

**Affiliations:** 1 Department of Health, Human Performance, and Recreation, Robbins College of Health and Human Sciences, Baylor University, Waco, TX 76706, USA; 2 Department of Human Sciences and Design, Robbins College of Health and Human Sciences, Baylor University, Waco, TX 76706, USA; michelle_l_law@baylor.edu

**Keywords:** amino acids, pre-clinical, mTOR, skeletal muscle, atrophy, inflammation

## Abstract

Cancer cachexia (CC) is a complex syndrome of bodily wasting and progressive functional decline. Unlike starvation, cachexia cannot be reversed by increased energy intake alone. Nonetheless, targeted nutritional support is a necessary component in multimodal syndrome management. Due to the highly catabolic nature of cancer cachexia, amino acid supplementation has been proposed. Interestingly, leucine has been found to increase protein synthesis and decrease protein degradation via mTORC1 pathway activation. Multiple pre-clinical studies have explored the impact of leucine supplementation in cachectic tumor-bearing hosts. Here, we provide an overview of leucine’s proposed modes of action to preserve lean mass in cachexia and review the current pre-clinical literature related to leucine supplementation during CC. Current research indicates that a leucine-rich diet may attenuate CC symptomology; however, these works are difficult to compare due to methodological differences. There is need for further pre-clinical work exploring leucine’s potential ability to modulate protein turnover and immune response during CC, as well as the impact of additive leucine on tumor growth.

## 1. Introduction

Cancer cachexia (CC) is described as a multifactorial syndrome in which there is ongoing loss of skeletal muscle and fat mass, leading to progressive functional decline [1]. This condition cannot be entirely reversed by traditional nutritional support. Oftentimes, CC reduces the tolerability and effectiveness of cancer therapies while also causing profound fatigue and weakness; moreover, it decreases both life expectancy and quality of life [2,3]. Due to the complex nature of this syndrome, treatment must be multimodal. CC is catabolic in nature; thus, nutritional supplementation with leucine has been posed as a coadjuvant treatment [4,5]. This branched-chain amino acid (BCAA) exerts downstream effects on muscle protein synthesis, culminating in the promotion of muscle protein anabolism. The effect of leucine supplementation in CC has been examined in recent pre-clinical studies, but a consensus has yet to be established. This review seeks to provide an overview of leucine’s proposed modes of action to preserve lean mass in cachexia and review the current pre-clinical literature related to leucine supplementation during CC.

## 2. Cancer Cachexia

CC is a syndrome of progressive bodily weight loss, leading to decreased quality of life and life expectancy. A common comorbidity in cancer patients, CC occurs in 30–90% of cancer cases [6,7,8]. It is most associated with cancers of the lung, liver, and gastrointestinal tract, such as gastric, pulmonary, pancreatic, esophageal, hepatic, and colorectal cancers [9]. A CC diagnosis can be made when body-weight loss is greater than 5% within six months, when body mass index (BMI) is less than 20 kg/m^2^ in combination with body weight loss of greater than 2%, or when the appendicular skeletal muscle index is consistent with sarcopenia and weight loss is greater than 2% [10].

Tumor-derived inflammation and the resulting systemic inflammation are understood to be the catalyst of CC. During cancer, systemic inflammation is caused by the release of cytokines from malignant tissue, inflammatory mediators, and activated immune cells [11]. This inflammation leads to metabolic chaos, resulting in muscle wasting and fat depletion [12]. Not only muscle and fat, but organs such as the heart, liver, and brain, are also impacted by this systemic inflammation [11,13,14,15,16,17]. Cancer negatively impacts the body’s ability to control normal energy balance, as many cancer patients become hypermetabolic [18,19,20,21]. Tumor metabolism, inflammation, and anti-cancer therapies may increase resting energy expenditure while also leading to decreased energy intake through loss of appetite or decreased ability to consume nutrients [22,23,24,25]. The tumor has its own metabolic rate, independent of the host. It competes for use of bodily fuel and substrates for biosynthetic processes [26]. Increased rates of whole-body glycolysis, gluconeogenesis, fatty acid cycling, as well as futile cycling, have all been observed during cancer and are thought to contribute to metabolic dysregulation [27,28,29,30]. Anorexia, nausea, and vomiting are common side effects of chemotherapy. These symptoms often lead to appetite loss and/or reduced food intake. Chemotherapy may also have direct implications on the metabolism [31]. This dangerous combination contributes to the negative energy balance observed during CC.

An expanding body of evidence suggests that the central nervous system (CNS) may be a key mechanistic driver in the pathogenesis of cachexia through its recognition of inflammation [32,33,34]. Once inflammation is sensed, the CNS transmits this information to various organ systems that in turn emit responses. Thus, downstream changes in metabolism are evoked. In this way, the CNS acts as an amplifier of peripheral inflammation. For acute threats, this is an adaptive pathway, but it becomes problematic during chronic conditions, such as cancer. One of the triggered metabolic responses is increased muscle catabolism. Particularly, in response to the cytokines that enter the CNS, the hypothalamic–pituitary–adrenal axis promotes lipolysis in adipose tissue and proteolysis in skeletal muscle [33,35,36,37]. Cytokine recognition by the hypothalamus has been found to lead to anorexia, weight loss, and skeletal muscle atrophy [35].

An imbalance between protein synthesis and protein degradation, favoring degradation over synthesis, leads to muscle loss. During cachexia in humans, lipolysis and proteolysis are estimated to increase by 30–80% and 40–60%, respectively [38,39,40]. Increased catabolism is thought to be mediated by the upregulation of the ubiquitin–proteasome system (UPS) and autophagy pathways [41,42,43]. The UPS is one of the body’s major proteolytic systems that selectively controls protein degradation [44,45,46]. Autophagy, on the other hand, is an intracellular pathway for bulk degradation of proteins, lipids, sugars, and nucleic acids by lysosomes [47,48,49,50]. These pathways can become overactive through metabolic, inflammatory, or hormonal stress signaling, as is visible in the case of CC [47,51]. The literature detailing the association between catabolism and CC is vast, this is but a brief mention of its involvement.

Currently, there is no standardized treatment protocol for CC. Due to the complex nature of this syndrome, a multimodal treatment approach is necessary [52,53,54]. Anamorelin, a ghrelin receptor agonist thought to help treat CC by improving appetite and increasing serum insulin-like growth factor-1, was recently approved in Japan and may prove to be a component in the multimodal treatment of CC [55]. Nonetheless, therapeutic options remain very limited. In stark contrast to starvation, increased energy intake alone does not correct CC because of the inflammatory response elicited by tumor and host tissues, which increases energy expenditure and favors protein catabolism. Even so, malnutrition, which is a common occurrence in cancer patients [56], is associated with poorer outcomes [57]. Cancer patients often have reduced calorie intake, caused by various factors including gastrointestinal tract obstruction due to tumor presence, general feelings of sickness and fatigue, decreased appetite, and/or adverse cancer or anticancer medication symptoms, such as nausea, vomiting, and diarrhea [58,59]. When at all possible, the correction of these conditions is important to increase caloric consumption amongst CC patients. Dietary counseling and nutritional support are key elements of multimodal CC treatment [60,61].

Importantly, adequate protein intake is required in CC because maintenance of skeletal muscle mass requires amino acid availability. Even small decreases in amino acid availability can be determinantal, changing the rate of protein synthesis and/or protein degradation and thereby resulting in muscle loss. No standardized protocol for minimizing skeletal muscle loss in this patient population currently exists. Many cancer patients are encouraged to increase their protein intake beyond the Recommended Dietary Allowance for adults, which is 0.8 g/kg/day. The European Society for Parenteral and Enteral Nutrition (ESPEN) recommends cancer patients ingest 1.0 to 1.5 g/kg/day of protein [62]. In addition to increased total protein intake, supplementation with BCAAs (leucine, isoleucine, and valine) as part of the multimodal treatment of CC has been proposed on multiple occasions [4,63,64,65]. Particularly, leucine has been found to enhance protein synthesis and decrease proteolysis independent of other BCAAs. In seminal work, Anthony et al. demonstrated leucine’s ability to enhance skeletal muscle protein synthesis through insulin-dependent, as well as insulin-independent, mechanisms [66,67]. Thus, nutritional supplementation with leucine has been explored as a potential additive treatment in the management of CC.

## 3. Role of Leucine in Muscle Metabolism

Muscle protein is in a constant state of turnover, as protein synthesis and protein degradation occur continuously. The “anabolic state” refers to a net gain of muscle protein, as the rate of muscle protein synthesis exceeds the rate of muscle protein breakdown. Conversely, the “catabolic state” refers to a net loss of muscle protein, as the rate of muscle protein breakdown exceeds the rate of muscle protein synthesis. Muscle protein is comprised of twenty amino acids, all of which are necessary for the synthesis of new muscle protein. Nine of these amino acids are considered essential, as they cannot be endogenously produced in sufficient quantities to meet the body’s needs and must be acquired through dietary protein sources. The BCAAs, three of the nine essential amino acids, are of considerable importance for muscle protein metabolism [68,69]. Leucine in particular is thought to be an anabolic mediator of protein metabolism [70,71,72].

**Figure 1:**
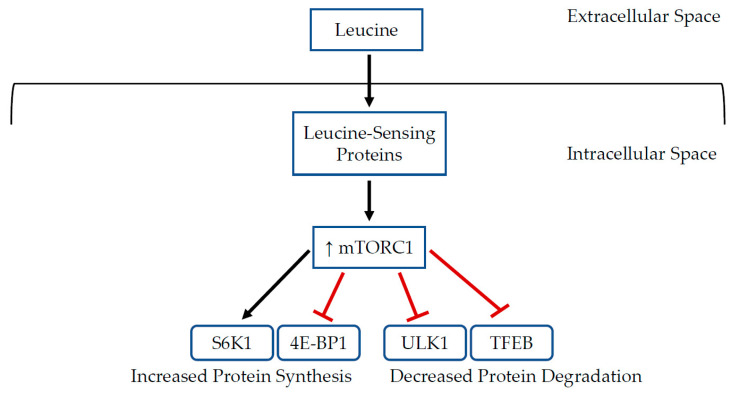
Schematic representation of leucine-induced activation of mTORC1 to promote anabolism and suppress catabolism.

As is well established, the mammalian target of rapamycin complex 1 (mTORC1) is an important regulator of cell growth and metabolism [73,74]. When activated, mTORC1 promotes anabolism and inhibits catabolism. Protein synthesis is stimulated by the direct phosphorylation and activation of the ribosomal protein S6 kinase 1 (S6K1) and inhibition of the eukaryotic initiation factor 4E binding protein 1 (4E-BP1) by the mTORC1 complex, resulting in enhanced mRNA translation and increased ribosomal protein levels [73,75,76,77]. Catabolism is suppressed via direct phosphorylation and inhibition of the transcription factor EB (TFEB) and unc-51-like autophagy activating kinase 1 (ULK1), thus suppressing lysosomal degradation and autophagy [76,78,79,80,81,82]. Activity of mTORC1 is influenced by multiple signaling molecules, including amino acids [76,83,84,85]. Particularly, leucine is known to influence mTORC1 activity [86,87,88]. During states of leucine abundance, leucine sensing proteins are activated via multiple mechanisms, which then cause recruitment and subcellular localization of mTORC1 complex proteins, leading to its activation and upregulation of protein translation (Figure 1). For further details regarding the complexities of leucine-mediated mTORC1 activation and signaling, the reader is referred to several excellent reviews [76,85,88,89,90,91,92].

Acute leucine supplementation in young and elderly human populations has been found to increase muscle protein synthesis [93,94,95,96,97,98]. Limited works explore leucine supplementation during CC. Currently, all data specifically exploring the relationship between leucine supplementation and CC symptomology attenuation seem to remain solely in pre-clinical animal models and will be described in this review.

## 4. Role of Leucine in Immune Function

The mammalian target of rapamycin (mTOR) is an important regulator of immune function, as has been established through experimental work with rapamycin, a macrolide inhibitor of mTOR [99,100]. mTOR is thought to be a signaling hub that senses and integrates information from the immune microenvironment to organize responses related to cell growth, proliferation, and death [99,100,101]. In recent years, mTOR was identified as a major regulator of adaptive immunity, as described in literature focused on memory CD8+ and CD4+ T-cell differentiation and human dendritic cell development [102,103,104,105]. The mTOR signaling pathway also plays a role in innate immunity, thought to direct effector response after being triggered by the activation of innate immune cells, such as innate-like natural killer cells, monocytes, neutrophils, mast cells, macrophages, and dendritic cells [106,107,108].

Evidence suggests that mTOR pathway signaling plays a role in the regulation of pro- and anti-inflammatory cytokines [109]. Inhibition of mTORC1 by rapamycin during Toll-like receptor stimulation has been shown to increase the expression of pro-inflammatory cytokine IL-12 via enhanced NF-κB signaling and decrease the expression of anti-inflammatory IL-10 via suppressed STAT3 signaling [110]. Suppression of mTOR signaling has also been reported to increase the expression of other pro-inflammatory cytokines such as TNFα, IL-23, and IL-6 in human monocytes and myeloid dendritic cells [111,112,113,114]. Nonetheless, some literature suggests that positive crosstalk between mTOR and NF-κB occurs during states of inflammation [115,116,117,118]. These results suggest that the role of mTOR signaling in the regulation of inflammation is complex and deserves further attention.

Based upon the potential anti-inflammatory action of mTOR pathway signaling, BCAA feeding may result in reduced inflammation during states of disease or muscle damage [119,120]. Thus, leucine may serve as a potential anti-inflammatory agent through its influence on the mTOR pathway [121,122,123]. However, how leucine specifically mediates inflammatory signaling through mTOR activation needs to be further explored. The limited pre-clinical data exploring the relationship between leucine supplementation and CC inflammation attenuation will be described in this review.

## 5. Methods

Due to the role of leucine in activating muscle protein synthesis and possibly modulating inflammation, the objective of this review was to identify and evaluate current literature exploring the impact of leucine supplementation on CC outcomes in animal models. A search of PUBMED and Medline databases was conducted. Papers found using the search terms “cancer cachexia” and “leucine supplementation” published between 2001 and 2021 were included. In vitro studies, review articles, and papers that did not include leucine supplementation during CC were excluded from this review. A total of 29 studies were initially retrieved and included as potentially relevant articles. Articles were then screened and excluded according to relevance. Of these publications, 14 were considered relevant for the purposes of this review and 15 were excluded due to lack of fit.

## 6. Results

### 6.1. Skeletal and Cardiac Muscle Effects

The majority of current literature focuses on the potential pro-anabolic and anti-catabolic effects of leucine supplementation during CC [124,125,126,127,128,129,130,131,132,133,134]. As previously stated, increased proteolysis via the UPS and autophagy pathways is a well-founded mechanism of muscle wasting during CC [135,136,137]. Early evidence suggesting that leucine supplementation may favorably impact protein turnover during CC was established in male Walker-256 tumor-bearing rats [126]. A leucine-rich diet (15% protein plus 3% leucine) reduced loss of lean body mass and gastrocnemius mass, as well as loss of myosin heavy chain content [126]. In a similar work, Ventrucci et al. demonstrated that leucine-rich feeding (15% protein plus 3% leucine) of female Walker-256 tumor-bearing rats modulated UPS activation [132]. Supplemented tumor-bearing rats demonstrated decreased proteasome subunit expression and slightly higher protein synthesis rates compared to their non-supplemented counterparts [132]. Work by Cruz et al. showed that leucine-rich feeding (18% protein plus 4.6% leucine) of Walker-256 tumor-bearing rats attenuated protein degradation and improved protein synthesis [124]. Similar to other studies, decreased proteasome subunit expression and increased muscle protein content were observed in the leucine group [124]. Using the C26 tumor-bearing CC model, Peters et al. found that leucine supplementation may preserve muscle mass in a dose-dependent manner [128]. Although both leucine groups exhibited some preservation of skeletal muscle mass relative to the non-supplemented tumor-bearing group, male tumor-bearing mice in the higher leucine group (14.8% leucine per gram protein) demonstrated greater attenuation of gastrocnemius and tibialis anterior muscle loss compared to the lower leucine tumor-bearing group (9.6% leucine per gram protein). Nonetheless, neither leucine group exhibited changes in markers of muscle protein degradation (mRNA Murf and mRNA Atrogin) and protein synthesis rates were not measured [128]. In a recent publication using male Walker-256 tumor-bearing rats, Viana et al. observed that a leucine-rich diet (18% protein plus 3% leucine) improved muscle strength and behavior performance, maintained body weight, fat, and muscle mass, and decreased MuRF-1 and proteasome 20S subunit expression [134]. Leucine supplementation did not affect muscle cross-sectional area but did increase total muscle protein concentration. Even so, the authors note that no association between leucine feeding and muscle oxidative capacity, inflammation status, or walking test performance was found [134].

The impact of leucine-rich feeding on cellular metabolism during CC has been explored [125,133]. Viana et al. used proton nuclear magnetic resonance (^1^H NMR) to evaluate the impact of leucine feeding (18% protein plus 3% leucine) on the metabolomic profile of a tumor-bearing host [133]. Evaluating the serum of Walker-256 tumor-bearing female rats, butyrate metabolism and ketone body metabolism appeared to be the two main pathways that were impacted by leucine feeding. These findings suggest that, since leucine is a ketogenic amino acid, increased leucine may provide not only increased substrate for protein synthesis but also for ketone production, and serves as an alternative fuel source, especially in light of decreased glucose availability due to the presence of the tumor. Leucine-rich feeding also resulted in lower levels of tryptophan and lactate, suggesting a decreased hypermetabolic state [133]. In a more recent publication, Cruz et al. examined the impact of leucine-rich feeding (18% protein plus 3% leucine) on the metabolomic state of skeletal muscle during CC [125]. Leucine supplementation of male Walker-256 tumor-bearing rats modulated pathways that favored mitochondrial biogenesis in skeletal muscle tissue, thus maintaining energy production. The expression of mitochondrial proteins related to oxidative phosphorylation was also preserved [125].

The combination of a leucine-rich diet with other potential CC treatment modalities has also been explored [127,129,130]. Using the C26 tumor-bearing CC model, van Norren et al. explored the effect of combined nutritional supplementation (high protein, leucine (1.6% leucine), and fish oil) compared to single-compound supplementation. Combined dietary supplementation was found to reduce body weight loss, attenuate tibialis anterior muscle wasting, and improve skeletal muscle functional performance. This relationship did not exist when dietary components were explored in isolation. It is important to note that the percent leucine used in this study was lower than previously mentioned works (1.6% vs. 3–4% leucine), which may be a potential reason why leucine supplementation alone did not have an effect [127]. When physical exercise and a leucine-rich diet (18% protein plus 3% leucine) were implemented in very young male Wistar rats with Walker-256 tumors, minimized muscle protein degradation and preserved muscle myosin content were noted [130]. Similarly, short-term light aerobic exercise combined with a leucine-rich diet (18% protein plus 3% leucine) resulted in reduced tumor weight and improved protein metabolism [129]. A leucine-rich diet combined with fish oil supplementation was found to improve tumor-induced hypercalcemia in male C26 tumor-bearing mice [138]. This is important, as hypercalcemia can result in muscle weakness and cardiac arrhythmias [139,140]. As stated earlier in this review, multimodal therapy is likely most efficacious for treatment of CC, as this is a complex, multifactorial syndrome. Therefore, the additive effect of leucine with other treatments targeting diverse mechanisms deserves further attention.

As previously mentioned, CC is a multi-organ syndrome. In addition to its negative impact on skeletal muscle, cachexia detrimentally impacts other organs, such as the heart. Though research regarding the cachectic heart and leucine supplementation is limited, we identified one pre-clinical study that explored this topic. In this study, Walker-256 tumor-bearing rats fed a leucine-rich diet (18% protein and 3% leucine) displayed attenuated cardiomyocyte proteolysis, heart damage, and apoptosis [131]. Nonetheless, leucine feeding was not observed to affect reduced left ventricular thickness [131].

### 6.2. Inflammatory Effects

A few of the identified studies focus on the potential positive immune-modulatory effects of leucine supplementation during CC [124,138,141]. Faber et al. used male C26 tumor-bearing mice to examine the impact of a specific nutritional combination (high protein, fish oil, leucine, and oligosaccharides), and its individual components, on inflammatory status and immune function during CC [141]. Combined supplementation of all components, but not individual ingredients independently, resulted in increased contact hypersensitivity responsiveness (measured by the level of ear swelling after hapten challenge with oxazolone solution topical applied to ear pinnae), decreased total n-6 content in cell membranes of splenocytes, reduced plasma levels of pro-inflammatory cytokines (IL-6, TNF-α, and PGE_2_), and a strong trend towards improved immune response was observed [141]. No effect on relative number of granulocytes, monocytes, or T cells was observed [141]. Plas et al. explored inflammatory mediator levels in male C26 tumor-bearing mice subjected to leucine-rich feeding combined with fish oil supplementation [138]. Plasma PGE-2 and tumor PTHrP levels were reduced in tumor-bearing animals fed an enriched diet [138]. Cruz et al. demonstrate that leucine-rich feeding (18% protein plus 4.6% leucine) of Walker-256 tumor-bearing rats resulted in an earlier increase in anti-inflammatory cytokines, including IL-4 and IL-10 [124]. These results suggest that leucine-rich feeding of a tumor-bearing host may elicit anti-inflammatory effects. Nonetheless, most of these studies utilized combined therapies, so leucine-specific effects are difficult to establish. More research is necessary to explore the potential relationship between leucine-rich feeding and inflammation mitigation.

### 6.3. Tumor Growth Effects

Though evidence is limited, recent pre-clinical evidence suggests that a leucine-rich diet may increase the rate of tumor growth. Two studies showed long-term leucine supplementation promoted bladder cancer development in rats treated with a known bladder carcinogen [142,143]. In research specific to CC, after receiving Panc02 cell injection, overweight and lean male mice supplemented with 5% percent leucine exhibited enhanced pancreatic tumor growth compared to their non-supplemented counterparts [144]. It is important to note that these animals were 6 to 8 weeks of age at study initiation but were not sacrificed until 27 weeks of age. These mice were significantly older and fed a diet higher in leucine (5% vs. 1.6–4% leucine) than other studies discussed above. In contrast, a previously mentioned work by Salomão and Gomes-Marcondes demonstrated decreased tumor burden [129]. Rodent age difference, tumor type, and other inconsistent methodological parameters, including leucine dosing and length of supplementation, make head-to-head comparison difficult. Further research exploring tumor growth during leucine supplementation is needed to determine safety. A brief description of each of the 14 included studies can be found in Table 1.

## 7. Discussion

Out of 14 included pre-clinical CC studies, 13 suggest that leucine-rich feeding may be a beneficial additive treatment for CC. The outlying study demonstrated increased tumor burden [144]. Current pre-clinical research that shows positive implications of leucine supplementation indicate that it may reduce skeletal muscle loss (via preserved protein synthesis and decreased protein degradation) [124,125,126,127,128,129,130,132], attenuate cardiac dysfunction [131], improve immune competence [141], preserve energy production capacity [125], and decrease inflammation [124,141]. A few of these studies are complicated by the inclusion of various anti-cachectic modalities, some of which report that CC symptomology attenuation was only achieved with a combined approach instead of leucine supplementation alone [127,129,130,138,141].

Currently, all literature that specifically examines the application of leucine-rich feeding during CC is limited to pre-clinical rodent studies. Though it is very beneficial for various reasons, pre-clinical cachexia research has inherent limitations. Variability exists between models, such as mechanism of cachexia development (cancer cell injection, carcinogen exposure, tumor grafting, etc.), age and sex of host, type and strain of rodent, rate of cachexia development, tumor location (ectopic vs. orthotopic) and burden, whether or not the tumor is metastatic, and use of anticancer medication [145,146,147]. This variability makes head-to-head comparison of pre-clinical CC research difficult, while also limiting the translatability to the human population. The limited CC pre-clinical literature exploring the application of leucine supplementation was completed using the Walker-256, C26, and Panco02 models [124,125,127,128,129,130,131,133,134,138,144], making study comparison difficult. For example, the Walker-256 model typically uses 13-week-old Wistar rats, whereas the C26 model uses 6- to 8-week-old CD2F1 or BALB/c mice [145]. Further, all current pre-clinical literature related to leucine supplementation in the context of CC has been performed using either male or female rodents. No study has used both sexes simultaneously. Thus, no sex differences can be explored. Another important consideration when examining animal research is interspecies differences in protein metabolism. Rodents have a higher rate of protein turnover compared to humans, estimated to be approximately 10 times faster [148,149,150]. In summation, these factors complicate the synthesis of current work and make overall interpretation of outcomes difficult.

Limited research suggests leucine supplementation may enhance tumor growth [142,143,144]. Currently, there is insufficient evidence to establish a cause–effect relationship. Even so, this possibility discourages the implementation of a leucine-rich diet in CC patients at present. More standardized rodent work is needed to further explore the safety and efficacy of leucine supplementation in the context of CC.

## 8. Conclusions

In this review, leucine supplementation in rodent models was discussed within the scope of CC. Current research indicates that a leucine-rich diet may attenuate CC symptomology; however, these works are difficult to compare due to methodological differences. There is need for further pre-clinical work exploring leucine’s potential ability to modulate protein turnover and immune response during CC, as well as the impact of additive leucine on tumor growth. If the safety of leucine supplementation is confirmed, prospective human clinical trials are needed. Further work is necessary to determine whether leucine supplementation may be a beneficial additive treatment for CC patients.

## Figures and Tables

**Table 1:** Leucine supplementation studies in animal models of cancer cachexia. In dietary details, C—control diet and E—experimental diet.

Author(s)	Animals	Dietary Details	Experimental Protocol	Major Effect(s)
Cruz et al., 2017 [124]	Walker-256 tumor model, female Wistar rats (*n* = 72, 90 days old, weighing 180–200 g)	C = 18% protein E = 18% protein + 3% leucine	12 groups according to Walker-256 status, tumor growth period, and diet	Attenuated skeletal muscle and protein content loss
Cruz et al., 2020 [125]	Walker-256 tumor model, male Wistar rats (*n* = 72, 90 days old, weighing 350–380 g)	C = 18% protein E = 18% protein + 3% leucine	4 groups according to Walker-256 status and diet	Attenuated energy production
Faber et al., 2008 [141]	C26 tumor model, male CD2F1 mice (6 to 7 weeks old)	C = 12.6% protein E = 15.1% protein ± 1.6% leucine and/or fish oil, or 21% protein ± 2.1% leucine and/or fish oil	6 groups according to C26 status and diet (control, single nutrient additive, or combination)	Combined diet led to reduced inflammation and improved immune competence
Gomes-Marcondes et al., 2003 [126]	Walker-256 tumor model, male Wistar rats (*n* = 36, 25 days old)	C = 18% protein E = 15% protein + 3% leucine	4 groups according to Walker-256 status and diet	Attenuation of lean carcass mass and muscle myosin loss
Liu et al., 2014 [144]	Panco02 tumor model, male C57BL/6 mice (*n* = 88, 6 to 8 weeks old—diet initiation, 23 weeks of age—tumor injection)	C = ~16% protein E = ~16% protein + 5% leucine	At 6 to 8 weeks, 4 groups according to diet and calorie restriction. After 23 weeks, some mice were euthanized while the remainder were redistributed into 4 groups according to Panco02 status	Enhanced tumor growth
Peters et al., 2011 [128]	C26 tumor model, male CD2F1 mice (*n* = 38, 6–7 weeks old)	C = 8.7% of protein as leucine E = 9.6% or 14.8% of protein as leucine	4 groups according to C26 status and diet (low and high leucine feeding)	Reduced skeletal muscle wasting
Plas et al., 2019 [138]	C26 tumor model, male CD2F1 mice (6–7 weeks old)	C = 12.6% protein E = 15.1% protein ± 1.6% leucine and/or fish oil	53 groups according to C26 status and diet (control, single nutrient additive, or combination)	Combined diet reduced elevated plasma PGE-2 and PTHrP levels
Salomão et al., 2010 [130]	Walker-256 tumor model, male Wistar rats (*n* = 93, 21 days old)	C = 18% protein E = 18% protein + 3% leucine	At 21 days, 4 groups according to exercise and diet. After 60 days, rats were redistributed into 8 groups according to Walker-256 status	Exercise and leucine supplementation in conjunction led to decreased negative alterations in protein turnover
Salomão et al., 2012 [129]	Walker-256 tumor model, male Wistar rats (*n* = 80, 35 ± 2 days old)	C = 18% protein E = 18% protein + 3% leucine or 4% glutamine, or both	8 groups according to Walker-256 status, exercise, and diet	Exercise and leucine supplementation in conjunction led to decreased negative alterations in protein turnover and carcass nitrogen content
Toneto et al., 2016 [131]	Walker-256 tumor model, male Wistar rats (*n* = 20, 90 days old)	C = 18% protein E = 18% protein + 3% leucine	4 groups according to Walker-256 status and diet	Attenuated cardiac failure
van Norren et al., 2009 [127]	C26 tumor model, male CD2F1 mice (6–7 weeks old)	C = 12.6% protein E = 15.1% protein ± 1.6% leucine and/or fish oil	6 groups according to C26 status and diet (control, single-nutrient additive, or combination)	Reduced loss of carcass, skeletal muscle, and fat mass loss with leucine-rich diet alone, combined diet resulted in a greater reduction in cachectic symptoms and improved functional performance
Ventrucci et al., 2004 [132]	Walker-256 tumor model, pregnant female Wistar rats (*n* = 60, 45 days old)	C = 18% protein E = 15% protein + 3% leucine	6 groups according to Walker-256 status, diet, and pair feeding	Reduced 20S, 19S, and 11S proteasome content and increased protein synthesis
Viana et al., 2016 [133]	Walker-256 tumor model, female Wistar rats (*n* = 35, 90 ± 10 days old, weighing 265 ± 10 g)	C = 18% protein E = 18% protein + 3% leucine	4 groups according to Walker-256 status and diet	Alterations in 23 serum metabolites with no increase in tumor size
Viana et al., 2021 [134]	Walker-256 tumor model, male Wistar rats (*n* = 24, 12 weeks old)	C = 18% protein E = 18% protein + 3% leucine	4 groups according to Walker-256 status and diet	Improved muscle strength and behavioral performance, no impact on walking test, inflammation status, or muscle oxidative capacity
